# Myocardial perfusion is impaired in renal transplant and liver transplant patients

**DOI:** 10.1186/1532-429X-17-S1-O10

**Published:** 2015-02-03

**Authors:** Susie F Parnham, Jonathan M Gleadle, Darryl Leong, Suchi Grover, Craig Bradbrook, Richard J Woodman, Carmine De Pasquale, Joseph Selvanayagam

**Affiliations:** 1 Department of Renal Medicine, Flinders Medical Centre, Bedford Park, SA, Australia; 2 Population Health Research Insititute, Hamilton, ON, Canada; 3 Flinders Medical Centre, Bedford Park, SA, Australia; 4 School of Medicine, Flinders University, Bedford Park, SA, Australia; 5 Department of Cardiovascular Medicine, Flinders Medical Centre, Bedford Park, SA, Australia; 6 School of Medicine, Flinders University, Bedford Park, SA, Australia; 7 Flinders Centre for Epidemiology and Biostatistics, School of Medicine, Flinders University, Bedford Park, SA, Australia

## Background

Cardiovascular disease is a common cause of mortality post renal transplantation, often manifesting in patients with no known cardiac disease. The cardiac phenotype in these patients is not clearly defined. We hypothesised that myocardial perfusion reserve would be impaired in renal transplant recipients compared with hypertensive controls, and similar to liver transplant recipients.

## Methods

We conducted a prospective study of 25 renal transplant (RT) recipients, 8 liver transplant (LT) recipients without previous CKD history and 7 controls with hypertension (HT). The transplant recipients were asymptomatic and had no previous ischaemic heart disease or revascularisation or systolic heart failure. The pre-transplant workup of the RT and LT were negative for haemodynamically significant epicardial coronary artery stenosis. Diabetes mellitus history between RT, LT and HT controls were not statistically different. Myocardial function, late-gadolinium enhancement and first-pass perfusion was assessed semi-quantitatively at rest and under stress. The MPRI was calculated as the ratio of perfusion during adenosine-induced hyperemia to the rest perfusion. The RT and LT patients underwent whole-heart non-contrast magnetic resonance coronary angiography (MRCA) to assess the presence of proximal to mid epicardial coronary artery disease.

## Results

A total of 708 myocardial segments of RT group, 198 myocardial segments of LT and 204 myocardial segments of HT controls were analysed and compared using mixed linear modeling. Left ventricular mass index and biventricular functions were similar between the groups. Tacrolimus use was similar between RT and LT groups (84% vs 89% respectively, p= 0.65). The mean transmural myocardial perfusion reserve across all myocardial segments was significantly lower in the RT and LT groups compared to HT controls (1.24 ± 0.48 in RT versus 1.29 ± 0.42 in LT versus 2.02 ± 0.38 in HT controls, p< 0.001). Amongst the transplant patients, 7 RT and 4 LT patients had coronary artery stenosis >50% in at least one territory, and 1 RT patient and 1 LT patient had late Gadolinium enhancement suggesting sub-endocardial infarction.

## Conclusions

Asymptomatic renal transplant recipients have impaired myocardial perfusion independent of the degree of left ventricular hypertrophy or the rate of diabetes mellitus. The global reduction in myocardial perfusion in the renal transplant is similar to the liver transplant recipients, thus unlikely related to the previous CKD, and it is not fully explained by the presence of asymptomatic epicardial coronary artery disease.

## Funding

N/A.

**Figure 1:**
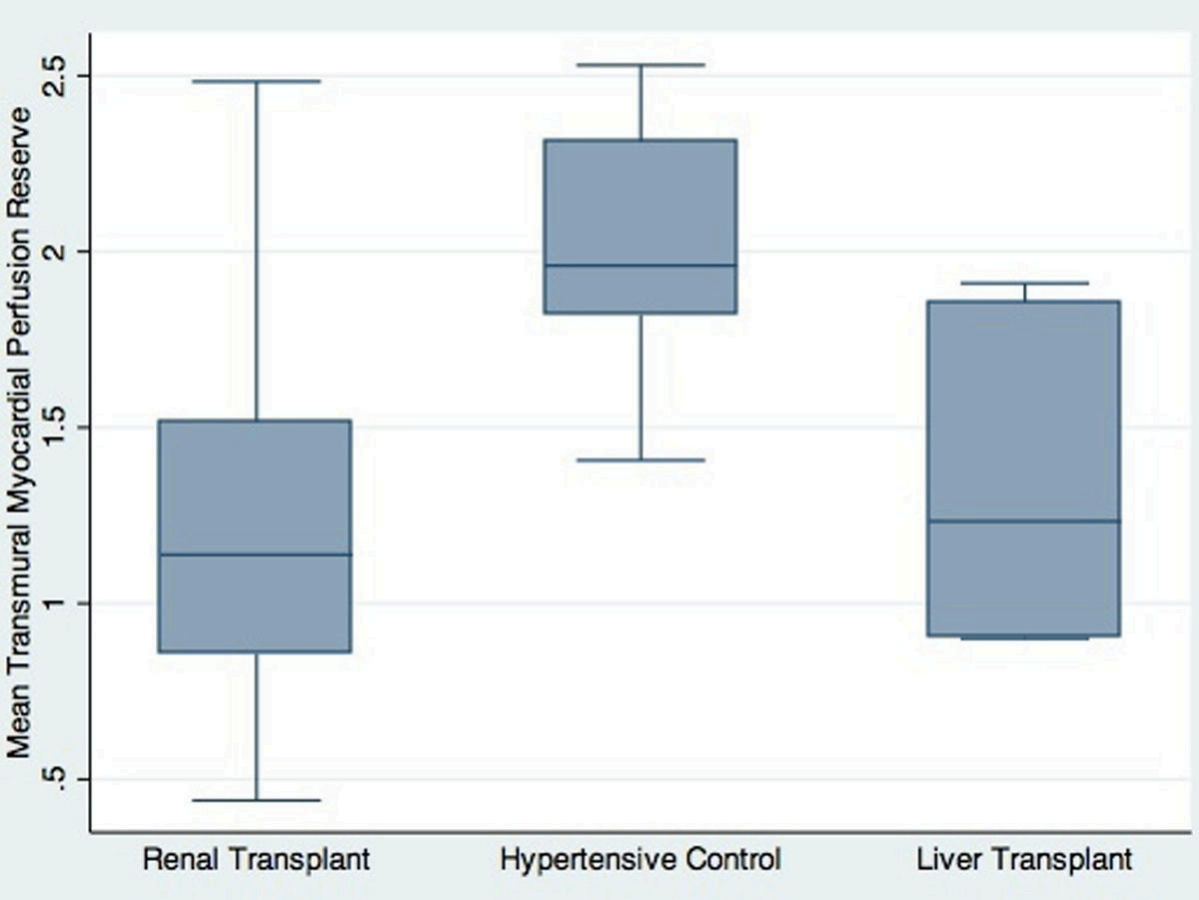
Mean transmural myocardial perfusion reserve.

